# Incorporating frailty to address the key challenges to geriatric economic evaluation

**DOI:** 10.1186/s12877-024-04752-5

**Published:** 2024-02-14

**Authors:** Joseph Kwon, Hazel Squires, Tracey Young

**Affiliations:** 1 Nuffield Department of Primary Care Health Sciences, University of Oxford, Radcliffe Primary Care Building, Radcliffe Observatory Quarter, Woodstock Road, OX2 6GG Oxford, England; 2 School of Health and Related Research, University of Sheffield, Regent Court (ScHARR), 30 Regent Street, S1 4DA Sheffield, England

**Keywords:** Frailty, Falls prevention, Economic model, Long-term outcomes, Societal outcomes, Equity

## Abstract

**Background:**

The multidimensional and dynamically complex process of ageing presents key challenges to economic evaluation of geriatric interventions, including: (1) accounting for indirect, long-term effects of a geriatric shock such as a fall; (2) incorporating a wide range of societal, non-health outcomes such as informal caregiver burden; and (3) accounting for heterogeneity within the demographic group. Measures of frailty aim to capture the multidimensional and syndromic nature of geriatric health. Using a case study of community-based falls prevention, this article explores how incorporating a multivariate frailty index in a decision model can help address the above key challenges.

**Methods:**

A conceptual structure of the relationship between geriatric shocks and frailty was developed. This included three key associations involving frailty: (A) the shock-frailty feedback loop; (B) the secondary effects of shock via frailty; and (C) association between frailty and intervention access. A case study of economic modelling of community-based falls prevention for older persons aged 60 + was used to show how parameterising these associations contributed to addressing the above three challenges. The English Longitudinal Study of Ageing (ELSA) was the main data source for parameterisation. A new 52-item multivariate frailty index was generated from ELSA. The main statistical methods were multivariate logistic and linear regressions. Estimated regression coefficients were inputted into a discrete individual simulation with annual cycles to calculate the continuous variable value or probability of binary event given individuals’ characteristics.

**Results:**

All three conceptual associations, in their parameterised forms, contributed to addressing challenge (1). Specifically, by worsening the frailty progression, falls incidence in the model increased the risk of falling in subsequent cycles and indirectly impacted the trajectories and levels of EQ-5D-3 L, mortality risk, and comorbidity care costs. Intervention access was positively associated with frailty such that the greater access to falls prevention by frailer individuals dampened the falls-frailty feedback loop. Association (B) concerning the secondary effects of falls via frailty was central to addressing challenge (2). Using this association, the model was able to estimate how falls prevention generated via its impact on frailty paid and unpaid productivity gains, out-of-pocket care expenditure reduction, and informal caregiving cost reduction. For challenge (3), frailty captured the variations within demographic groups of key model outcomes including EQ-5D-3 L, QALY, and all-cause care costs. Frailty itself was shown to have a social gradient such that it mediated socially inequitable distributions of frailty-associated outcomes.

**Conclusion:**

The frailty-based conceptual structure and parameterisation methods significantly improved upon the methods previously employed by falls prevention models to address the key challenges for geriatric economic evaluation. The conceptual structure is applicable to other geriatric and non-geriatric intervention areas and should inform the data selection and statistical methods to parameterise structurally valid economic models of geriatric interventions.

**Supplementary Information:**

The online version contains supplementary material available at 10.1186/s12877-024-04752-5.

## Background

The process of ageing encapsulates multidimensional changes that occur over the life course in both the physical and psychosocial spheres of an individual [1]. At the physical level, ageing is associated with gradual accumulation of diverse molecular and cellular damages [2]. These lead to progressive, generalised impairments in physical capacities such as muscle strength, mobility, and cognition [3–5], increased risks of chronic diseases [6], greater vulnerability to environmental challenges such as immuno-senescence [7], and ultimately death [2]. At the psychosocial level, ageing typically involves shifts in social roles and circumstances, including higher risks of financial stress, social isolation, and emotional toll of bereaving the loss of close relations. These psychosocial stressors can interact with physical vulnerabilities to induce poor mental health and further physical deconditioning [8].

These features of geriatric health bring challenges in implementing and evaluating geriatric clinical or public health interventions. The first major challenge is to account for the full consequences, including indirect and long-term outcomes, of seemingly minor stressor events experienced by older persons. Even a fall incurring no injury, for example, has been shown to be significantly associated with functional difficulties in older persons over the following two years [9]. How such an effect could occur is dynamically complex, likely working through several intermediary causal links within a feedback loop [10]: e.g., fall → loss of confidence in balance → activity curtailment → physical deconditioning → further decline in balance [11]. Evaluation of a geriatric health shock must therefore account not only for its direct impact but also for its indirect, long-term influences on diverse physiological, functional, and psychosocial systems.

The second challenge is to implement person-centred care, namely addressing the multidimensional health and non-health needs of each older person [1, 12]. Evaluating such care requires capturing a broad range of outcomes that are of importance to older persons, such as financial security, remaining productive in paid or unpaid roles, and social wellbeing; in other words, an outcome range broader than measures of health and healthcare costs alone [13–15]. In economic evaluations, i.e., the comparative analyses of alternative healthcare strategies in terms of costs and consequences, this would likely involve taking the societal perspective to evaluation [16].

A corollary to the complexity of needs at the individual level is the heterogeneity at the population level, particularly for public health interventions targeting a broadly defined population (e.g., adults aged 60 and over) rather than a narrow clinical patient group. The third challenge therefore consists in understanding the heterogeneous risks, capacities to benefit, and outcomes within the same demographic group (e.g., defined by age and sex). This heterogeneity can introduce priority setting challenges if the most vulnerable groups derive the least favourable effectiveness and cost-effectiveness outcomes [17, 18]. In the context of economic evaluation, this motivates the use of decisional criteria beyond cost-effectiveness, to incorporate equity considerations [19–21].

The concept of frailty has been proposed to capture the multidimensional and syndromic (i.e., not reducible to a specific disease or clinical diagnosis) nature of geriatric health and is thus useful for helping to address the above challenges to evaluation [22, 23]. In frail persons, a minor stressor event can trigger sudden and irreversible health changes, resulting in acute hospitalisations, nursing home admissions, and mortality [24–26]. There are two main types of frailty measures in the literature: phenotypic and cumulative deficit. The former tracks the presence of specific phenotypes that indicate vulnerability in multiple organ systems (e.g., unintentional weight loss, slow walking speed); it hence generates categorical measures [27]. The cumulative deficit measure tracks a group of deficits (at least 30) and calculates a multivariate frailty index between range 0–1 as a ratio between actual and potential numbers of deficits [28, 29]. Both measures aim to capture the holistic status of the geriatric patient and the interactions between diverse health deficits.

Decision modelling is a vehicle for economic evaluation that combines multiple epidemiological, intervention, and health economic evidence from diverse sources [30]. Decision models have several advantages over economic evaluations conducted alongside single clinical studies, such as the potential for incorporating long-term trajectories of disease risk factors, including that of frailty, and evaluating alternative scenarios [31]. To develop a structurally valid and credible decision model, the key features of disease epidemiology and intervention features should first be conceptualised based on stakeholder input and the academic literature [10, 32]. This conceptualisation should be free from constraints imposed by data availability and technical skillset of the modelling team. The conceptual model would then inform the structure and parameterisation of the final quantitative model using the available data and techniques.

This article aims to explore how incorporating a frailty measure (specifically, a multivariate frailty index) in a decision model can potentially address the above three challenges to geriatric economic evaluation. It proceeds first by presenting a conceptual structure of how a frailty measure can address the challenges, followed by a case study in parameterising an economic model of community-based falls prevention for older persons (aged 60 and over) [33]. This case study sought to translate the frailty-based conceptual structure to a quantitative model suitable for economic evaluation.

## Methods

### Conceptual structure

**Fig. 1:**
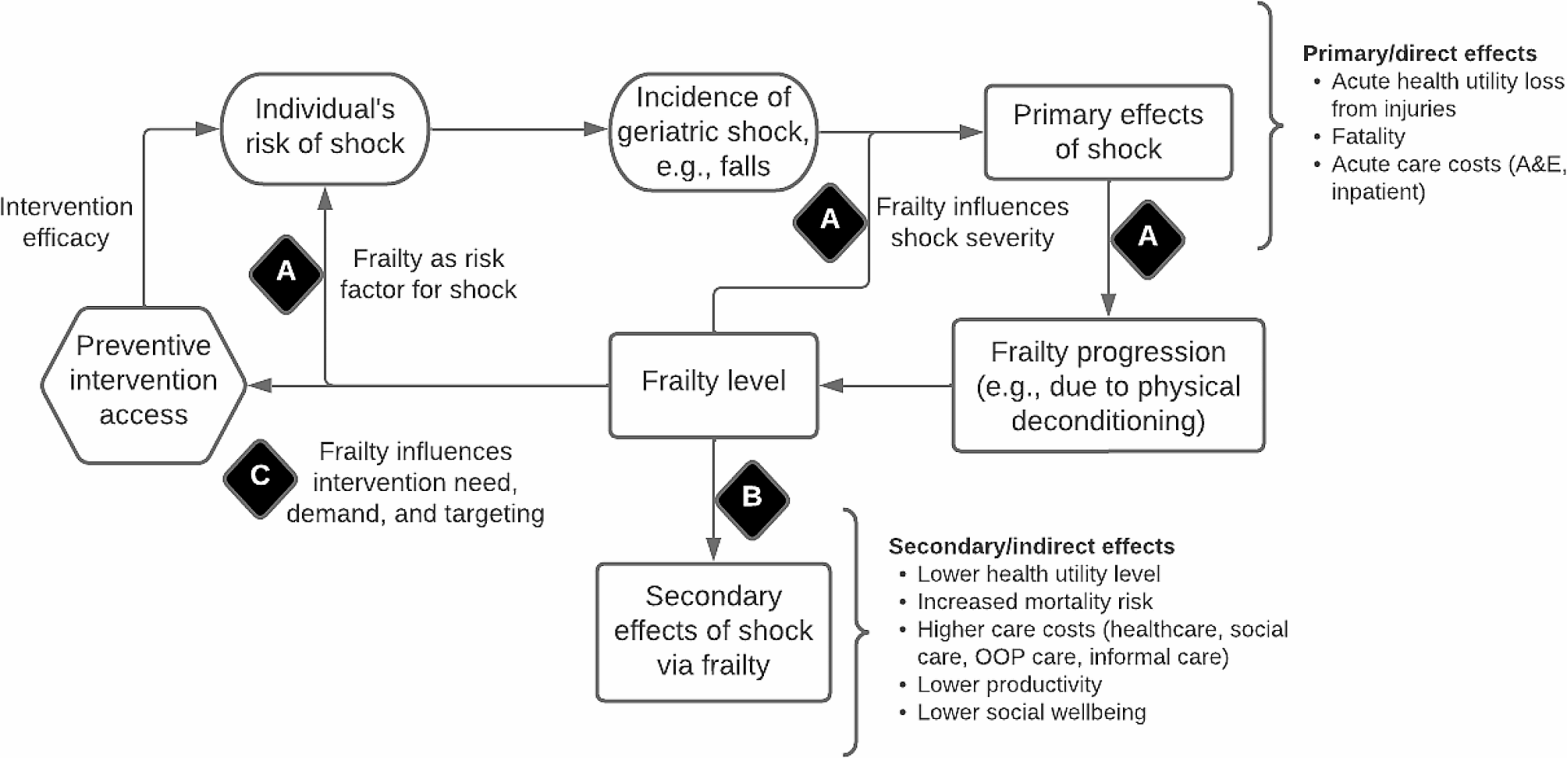
Relationship between falls and frailty: key associations A to C marked in black diamonds. Abbreviation: A&E: accident and emergency; OOP: out-of-pocket

Figure 1 shows the basic conceptual structure of the relationship between a geriatric shock and frailty. The first key association within this structure is the ‘shock-frailty feedback loop’, marked by the ‘A’ in a black diamond. A fall, as a case of geriatric shock, can have several primary or direct effects, including acute health utility loss from injuries, various acute care costs, and even fatality. Beyond these short-term effects, the fall can also induce medium- and long-term activity curtailment and physical deconditioning. These in turn worsen frailty [9], which subsequently increases the risk and severity of falls to complete the feedback loop [34, 35].

The second key association (marked ‘B’) concerns the secondary effects of falls propagated by the new frailty level and thus only indirectly associated with the initial shock. These effects include permanently lower health utility level and higher mortality risk as well as permanently higher care costs. These may be classified as ‘comorbidity’ health status and care costs, respectively, not because they are unrelated to the initial shock but because they are only indirectly related. Moreover, the secondary effects are likely to be intersectoral and cover a wide range of non-health outcomes, including lower productivity, higher out-of-pocket (OOP) care expenditure, and higher informal caregiver burden.

The third key association (marked ‘C’) concerns how the new frailty level influences subsequent intervention access. In the community-based falls prevention context, decision-makers may choose to prioritise prevention according to frailty category: see an example of such scheme in Sheffield, UK [36] wherein falls risk screening using quantitative timed-up-and-go (QTUG) test targeted those with moderate frailty. In the absence of a frailty score, decision-makers may prioritise through a related variable such as gait and balance impairments [37]. Frailty may also affect the demand of older persons for preventive activities such as group exercise [33]. Current UK guideline recommends preventive physical activity at all levels of frailty and dementia, as long as supervision by a trained professional is available [38]. This suggests that association C would be present at all stages of the frailty progression, even if intervention type and efficacy are moderated by the frailty level. The shape of the feedback loop would likely persist even after transition to institutionalised settings until death.

This article aims to show that parameterising one or more of the conceptual associations A to C involving frailty contributes to addressing the aforementioned challenges inherent in geriatric economic evaluation, namely: (1) accounting for indirect, long-term impacts of geriatric shocks; (2) incorporating a wide range of societal outcomes; and (3) accounting for heterogeneity within the same demographic group. Specifically, an economic model of community-based falls prevention [33] is used as a case study of how the conceptual structure was translated or parameterised into the final quantitative form to estimate the outcomes relevant to the decision problem. The rest of the Methods section summarises the data and statistical and modelling techniques used for parameterisation, while the Results section details the role of frailty in the parameterised model.

### Data: English longitudinal study of ageing

English Longitudinal Study of Ageing (ELSA) was chosen as a main data source because it collects a wide range of health, demographic, socioeconomic, and lifestyle variables relevant to older populations and tracks their longitudinal trajectories via individual identifiers [39, 40]. Particularly useful for this case study, ELSA also contains falls incidence and falls prevention service use data with which falls risk equations and service use patterns can be estimated using individual-level characteristics.

To date (May 2023), nine two-year interim ELSA surveys have been conducted starting from Wave 1 in 2002 to Wave 9 in 2018. The anthropometric and physical capacity (e.g., walking speed) variables were collected by nurse visits at even-number waves [41]. Among the nine waves, Waves 4 and 5 were used for parameterisation in this case study because Wave 4 contains the most comprehensive data regarding falls and falls prevention. Specifically, it is the only Wave with information on falls history in the previous *one year* rather than two years of survey interval. This variable is important because the NICE falls prevention guideline emphasises falls history in the previous 12 months [37]. Likewise, only Waves 2, 4 and 8 contain self-reported data on contact with falls prevention services (e.g., whether doctor/nurse tested balance and strength). More information on how ELSA was used for parameterisation is available elsewhere [42].

### Multivariate frailty index

A new 52-item multivariate frailty index was developed to suit the available data in ELSA. Care was taken to ensure that the index is broadly consistent in characteristics with previous indices used in frailty and falls prevention research [25, 34, 35, 43–45]. Table 1 shows the component items of the new and previous indices grouped into higher categories. The new index contained the five frailty phenotypes included in the Fried phenotypic measure [27]: slow walking speed; weak grip strength: significant weight loss; self-reported exhaustion; and low physical activity. It also contained the major falls risk factors (except for environmental fall hazards) highlighted by the NICE falls prevention guideline (p. 47) [37]: gait deficit; balance deficit; mobility impairment; visual impairment; cognitive impairment; and urinary incontinence. It did not contain falls as a component item to ensure that falls incidence does not increase frailty by default but only via association.

The number of deficits per individual was divided by the total possible number (52) to derive the index score. For the ELSA sample aged 60 and over (60+), the score ranged between 0 and 0.615 and had mean of 0.11 (standard deviation 0.09) for men and 0.13 (SD 0.10) for women. The scores were grouped into frailty categories– Fit, Mild, Moderate, and Severe– by a previously used method [25], with the cut-off levels at the 50th, 85th and 97th percentile values, respectively. The resulting score ranges were 0-0.10 for Fit, > 0.10–0.23 for Mild, > 0.23–0.37 for Moderate, and > 0.37 for Severe. For model parameterisation, the scores were multiplied by 100 to range 0-100. Visual plots had shown that the scores followed a lognormal distribution. The mean and SD for the lognormal distribution were hence obtained for each of the 280 subgroups, divided by age group (7 categories), sex (2), social deprivation quartile (4), and falls history (5). Table A1 in Supplementary Material presents the mean and SD by subgroup alongside further details on how the component items were selected.

### Statistical methods

The main statistical methods for parameterisation were multivariate logistic or linear regressions. Exceptions were instances when a dependent variable was significantly associated with only a few explanatory variables, in which case its subgroup-specific central statistics were used as parameters. The regressions were undertaken to estimate and quantify the conceptual associations displayed in Fig. 1. For example, the association between falls incidence and the rate of frailty progression and that between frailty and falls risk were estimated, after adjusting for further explanatory variables, to parameterise the falls-frailty feedback loop.

Several regressions were longitudinal in that the dependent variables were taken from ELSA Wave 5 and the explanatory variables from Wave 4. The range of potential explanatory variables for the regressions was limited to those incorporated in the simulation model described below. These variables were chosen based on a conceptual understanding of key factors influencing falls risk and prevention, such as cognitive impairment, abnormal gait/balance, and fear of falling; the conceptual model has been published elsewhere (see Appendix A of [33]). Final explanatory variables and their form (e.g., quadratic terms for age and frailty) were selected based on the combination that produced the lowest Akaike and/or Bayesian information criterion (AIC and BIC) values for the given dependent variable.

It should be noted that estimations were conducted for associative patterns rather than causal inference. Estimated regression coefficients were inputted into the simulation model to calculate the continuous variable value (from linear regression) or probability of binary event (from logistic) given simulated individuals’ characteristics. Equation (1) was used to calculate the probability of a binary event:

1 $$ \widehat{P\left(Event|X\right)}=\frac{EXP(\widehat{{\beta }_{0}}+\widehat{{\beta }_{1}}{X}_{i1}+\dots +\widehat{{\beta }_{k}}{X}_{ik})}{1+EXP(\widehat{{\beta }_{0}}+\widehat{{\beta }_{1}}{X}_{i1}+\dots +\widehat{{\beta }_{k}}{X}_{ik})}$$

$$ {X}_{ij}$$ describes the value of the explanatory variable or characteristic *j* for individual *i* and the event in question. $$ \widehat{{\beta }_{1}}$$ to $$ \widehat{{\beta }_{k}}$$ are the estimated logistic regression coefficients for the characteristics, and $$ \widehat{{\beta }_{0}}$$ the constant term. A negative coefficient below zero indicates negative association between the likelihood of event and the given explanatory variable, and vice versa. The variance-covariance matrices were stored for probabilistic sensitivity analysis.

**Table 1:** Characteristics of multivariate frailty indices used in previous frailty and falls prevention studies and in this study

	eFI [46]	BLSA FI [34, 43]	GLOW FI [35, 44]	ProAct65 + FI^1^ [45]	This study
Country	UK	China	Canada	England	England
Data source	Electronic health records	Cohort survey	Cohort survey	Cohort survey	Cohort survey
Total # of items	36	33^2^	34	40	52
Mean (SD)	Men: 0.13 (0.09) Women: 0.15 (0.10)	Men: 0.11 (0.10) Women: 0.14 (0.12)	Women only: 0.24 (0.13)	Both sex: 0.16 (0.11)	Men: 0.11 (0.09) Women: 0.13 (0.10)
Severity categories	[Fit] 0-0.12 (50%) [Mild] > 0.12–0.24 (35%) [Moderate] > 0.24–0.36 (12%) [Severe] > 0.36 (3%)	[1] 0-0.03 [2] > 0.03–0.10 [3] > 0.10–0.20 [4] > 0.20–0.50 [5] > 0.50	[Robust] 0-0.20 (43.9%) [Prefrail] > 0.20–0.35 (34.1%) [Frail] > 0.35 (22.1%)	[Non-frail] 0-<0.25 (81.5%) [Frail] > = 0.25 (18.5%)	[Fit] 0-0.10 (50%) [Mild] > 0.10–0.23 (35%) [Moderate] > 0.23–0.37 (12%) [Severe] > 0.37 (3%)
***Component items***					
Chronic diseases	(17) Anemia; Arthritis; AF; CBVD; CKD; Diabetes; Heart failure; Heart valve disease; Hypertension; Hypotension; IHD; Osteoporosis; PD; PVD; RD; Thyroid disease; Urinary system disease	(8) Arthritis; Cataract; CHD; Glaucoma; Hypertension; Stroke; Thyroid disease; TIA	(13) Cancer; Celiac disease; Chronic bronchitis; Crohn’s disease; Diabetes; Heart disease; High cholesterol; Hypertension; Multiple sclerosis; Osteoarthritis; PD; RA; Stroke	(15) Blood disease; Cancer; Digestive disease; Ear disease; Endocrine disease; Eye disease; Genitourinary disease; Heart disease; Infectious disease; Mental disease; MSKD; Nervous disease; RD; Skin disease; Other disease	(20) Angina; Arrhythmia; Arthritis; Asthma; Cancer; Cataract; Depression; Diabetes; DED; DKD; Glaucoma; Heart attack; Heart disease– other; Heart murmur; Hypertension; High cholesterol; Lung disease; MD; Osteoporosis; Stroke
Sensory/physical impairments and geriatric syndromes	(12) Hearing impairment; Visual impairment; Dizziness; Dyspnea; Falls; Foot problems; Fragility fractures; Peptic ulcer; Skin ulcer; Sleep disturbance; UI; Weight loss and anorexia	(5) Hearing problem; Use a hearing aid; Use a walking aid; Tremor; UI	(1) Unintentional weight loss	(2) Use a walking aid; Balance problems	(8) Seeing difficulties; Hearing difficulties; Slow walking speed;^3,4^ Balance problems;^4^ Weak grip strength;^3,4^ Weak leg strength;^4^ UI; Significant weight loss^3,4^
Cognitive impairment	(1) Memory and cognitive problems	(1) MMSE < 15			(1) Composite measure of cognitive problems across 4 tests of memory, mental speed and numeracy
Subjective symptoms and health status		(5) Lack of energy; Felt less useful; Don’t feel a lot of fun in life; Don’t feel very happy; Feel nothing to do	(6) Feels full of life; Has a lot of energy; Feels worn out; Feels tired; Self-rated health; Self-rated pain	(6) Feeling calm; Have a lot of energy; Feeling low; Social activity interfered by physical and emotional health; Self-rated health; Normal work interfered by pain	(4) Self-reported exhaustion;^3^ Self-rated health; Self-rated pain; Self-reported long-standing illness
Lifestyle risk factors				(2) Obesity (BMI > = 30); Low physical activity	(2) Low physical activity;^3^ Obesity
Activity limitation	(3) Any activity limitation; Housebound; Mobility and transfer problems	(14) ADL & IADL limitations	(12) ADL limitations	(14) ADL & IADL limitations	(15) ADL & IADL limitations
Healthcare contact	(2) Polypharmacy (5 + medications); Requirement for care		(2) Polypharmacy (5 + medications); Frequency of healthcare visit in past year	(1) Polypharmacy (6 + medications)	(1) Polypharmacy (5 + medications)
Social	(1) Social vulnerability				(1) Living alone

Abbreviation: ADL: activities of daily living; AF: atrial fibrillation; BLSA: Beijing Longitudinal Study of Aging; CBVD: cerebrovascular disease; CHD: coronary heart disease; CKD: chronic kidney disease; DED: diabetic eye disease; DKD: diabetic kidney disease; eFI: electronic frailty index; FI: frailty index; GLOW: Global Longitudinal Study of Osteoporosis in Women; IADL: instrumental activities of daily living; IHD: ischemic heart disease; MD: macular degeneration; MMSE: mini-mental status examination; MSKD: musculoskeletal disease; PD: Parkinson’s disease; PVD: peripheral vascular disease; RA: rheumatoid arthritis; RD: respiratory disease; SD: standard deviation; TIA: transient ischemic attack; UI: urinary incontinence

^1^ The frailty index was constructed using data from the randomised controlled trial ProAct65 + which compared group- and home-based falls prevention exercise to usual care in London, Nottingham and Derby [47].

^2^ The original index contained 35 items including falls and fracture [43]; the latter were taken out from index and used as outcomes in subsequent study [34].

^3^ Components of the frailty phenotypes proposed by Fried and colleagues [27].

^4^ These variables had more than 5% missing values which were imputed by multivariate single imputation.

### Simulation model of community-based falls prevention

**Fig. 2:**
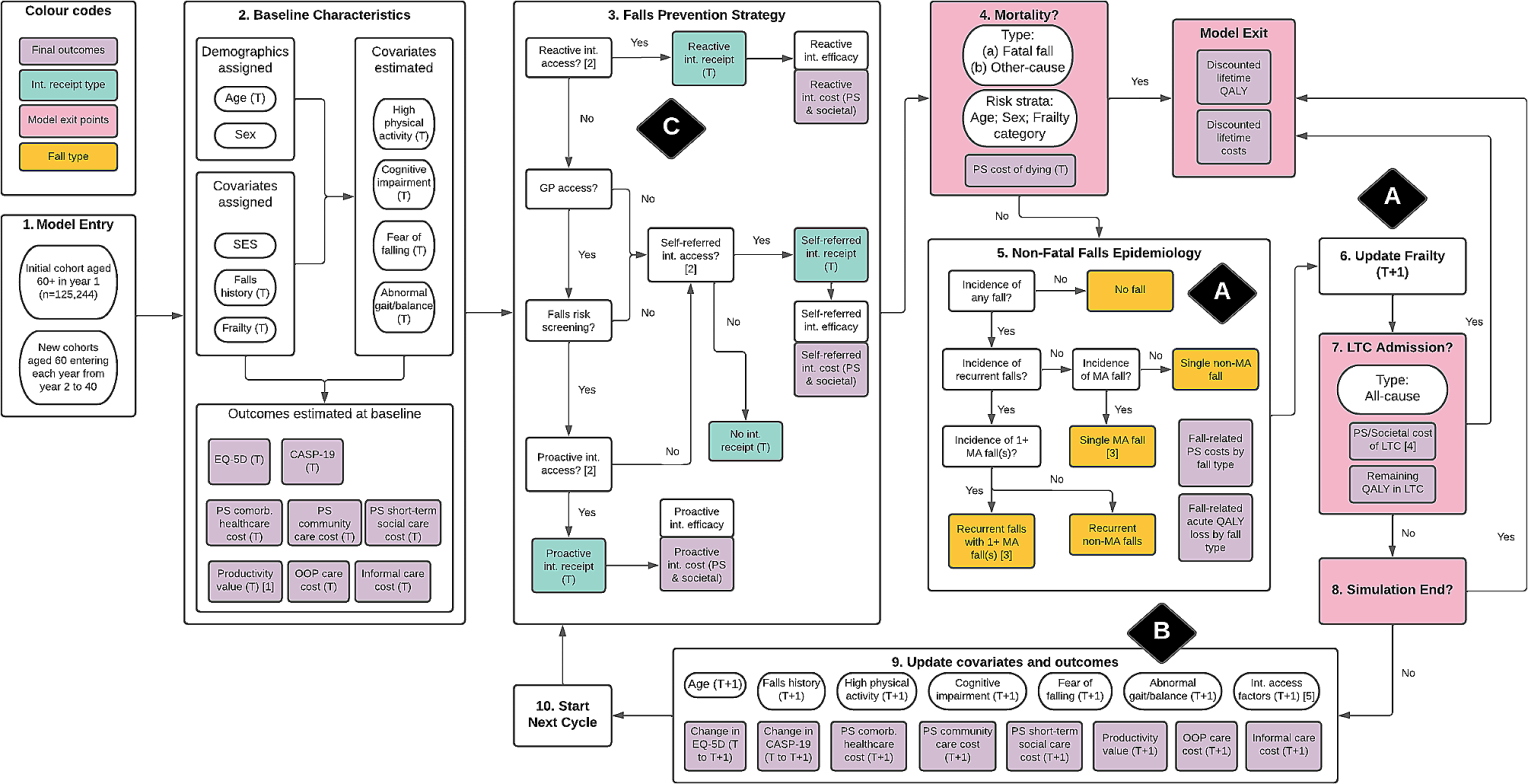
Model representation diagram: key associations A to C marked in black diamonds. Abbreviation: CASP-19: control, autonomy, self-realisation and pleasure, 19 items; Comorb.: comorbidity; Int.: intervention; LTC: long-term care; MA fall: fall requiring medication attention; OOP: out-of-pocket; PS: public sector; QALY: quality-adjusted life year; SES: socioeconomic status. Notes: [1] Includes paid employment and unpaid work [2]. Intervention access rates are functions of eligibility (determined by covariates such as falls history) and implementation factors (demand and supply capacity); these can be altered by intervention scenarios [3]. For those experiencing recurrent falls with 1 + MA fall(s), the probability for experiencing a second MA fall is applied; MA falls are subdivided into hospitalised and non-hospitalised MA falls [4]. The share of LTC cost incurred by public sector depends on individual’s SES quartile [5]. Probability of GP contact and demand for self-referred intervention are updated longitudinally

A discrete individual simulation (DIS) with annual cycles was developed to assess the cost-effectiveness of community-based falls prevention. The target population is community-dwelling adults aged 60 + in Sheffield, seen as being representative of urban UK local health economies. Figure 2 graphically represents the model including its covariates, falls prevention pathways, fall types, exit points, and final outcomes. Moreover, the key associations A to C conceptualised in Fig. 1 are similarly marked. The model was validated structurally, internally, and externally. The methods and results of conceptualisation, parameterisation, validation, and base case analysis of the model used here have been published in more detail elsewhere [33].

In the base case analysis, the model compared two intervention strategies: recommended care (RC) representing the recommendations by the UK falls prevention guidelines [37, 38, 48] versus usual care (UC) representing current practice in Sheffield. Both strategies involved three pathways operating in tandem: (i) reactive– wherein older persons who experienced a fall requiring medical attention are referred to rehabilitative interventions; (ii) proactive– initiated by older persons’ routine contact with care professionals at which those screened to be at high falls risk are referred to preventive interventions; and (iii) self-referred– wherein older persons enrol in an intervention (e.g., group exercise) without direct professional referral. RC and UC differed regarding the eligibility and implementation conditions under the three pathways. For example, in UC, only those *hospitalised* for a fall were referred to reactive intervention, as opposed to those receiving *any* medical attention for a fall under RC.

The base case analysis adopted the societal perspective under a 40-year time horizon. RC had 93.4% of being cost-effective versus UC at a cost-effectiveness threshold of £20,000 per QALY gained. RC increased productivity and reduced OOP care expenditure and informal caregiving cost versus UC, but these were outstripped by increases in intervention time opportunity costs and co-payments, respectively. RC also reduced inequality in incremental net health benefit in terms of socioeconomic status (SES) quartile.

The model parameterisation results are discussed below to illustrate how incorporating the frailty index addresses the key challenges to geriatric economic modelling.

## Results

### Simulation population characteristics

Table 2 summarises the characteristics of the simulated population at model entry.

**Table 2:** Baseline characteristics of simulated population

Mean age (SD)	70.4 (7.9)
Female (%)	53.8
SES quartile (%)^1^	
*Most privileged* *2nd* *3rd* *Most disadvantaged*	27.3 18.4 35.2 19.1
Falls history (%)	
*No falls history* *Single non-MA fall history* *Recurrent non-MA falls history* *Single MA fall history* *Recurrent falls with one or more MA fall history*	74.6 10.2 8.4 4.0 2.8
Mean frailty index (SD)	12.2 (9.9)
High physical activity (%)	17.3
Cognitively impaired (%)	20.5
Fear of falling (%)	6.8
Abnormal gait and/or balance (%)	28.0
In paid employment (%)	19.4
Engaged in unpaid work (%)	27.4
Paid for private care out-of-pocket (%)	3.4
Received informal care (%)	24.9

^1^ The SES variable combined education, wealth, and self-reported financial difficulty to form a composite score ranging from 3 to 12. The discrete numbers of the categorical SES variable produced uneven quartile sizes.

Abbreviation: MA fall; fall requiring medical attention; SD: standard deviation; SES: socioeconomic status

The contributions of frailty to geriatric economic modelling are described in the three sections below, a section for each of the key challenges. Each section describes how the key conceptual associations A to C in Figs. 1 and 2 were parameterised in the community-based falls prevention model.

### Accounting for indirect, long-term effects of shock

For this challenge, the conceptual association A, the shock-frailty feedback loop, plays an important role. The loop was parameterised as follows. First, the relationship between frailty and falls incidence was parameterised. Table 3 shows the coefficient estimates from the best-fit logistic regression for falls incidence between ELSA Waves 4 and 5. Importantly, frailty is positively and significantly associated with the risk (at a decreasing rate as shown by the negative coefficient for the quadratic term). Figure A1 in the Supplementary Material graphically illustrates the positive relation between falls risk and frailty category. The coefficient estimates were inputted into Eq. (1) shown in the Statistical Methods section when individuals entered box ‘5. Non-Fatal Falls Epidemiology’ in Fig. 2 at each model cycle to calculate their probabilities of falling (which were then annualised for the one-year model cycle length). When a fall occurred, the model assigned *acute* healthcare costs and QALY loss according to its severity which was determined by further regressions (detailed elsewhere [33]).

**Table 3:** Logistic regression for any fall incidence

*Dependent variable: Incidence of any fall between ELSA Waves 4 and 5* (*N* *= 6,205)*^*1*^		
Explanatory variables	Coefficient (SE)	*P*-value
Constant	0.067 (0.020)	< 0.001
Age	0.009 (0.004)	0.028
Female	0.187 (0.061)	0.002
Falls history one year prior to W4 survey^2^ (ref: No falls history)		
*Single non-MA fall*	0.845 (0.090)	< 0.001
*Recurrent non-MA falls*	1.654 (0.102)	< 0.001
*Single MA fall*	0.657 (0.141)	< 0.001
*Recurrent falls with MA*	0.974 (0.166)	< 0.001
Frailty (0-100)	0.049 (0.010)	< 0.001
Frailty^2	-0.0007 (0.0002)	0.002
Fear of falling	0.279 (0.125)	0.026
Abnormal gait/balance	0.148 (0.084)	0.079

^1^ Sample restricted to those interviewed in both ELSA Waves 4 and 5.

^2^ ELSA Wave 4 differs from other Waves in asking about falls incidence in the previous one year of survey, rather than since the previous survey two years ago.

Abbreviation: ELSA: English Longitudinal Study of Ageing; MA fall: fall requiring medical attention; ref: reference; SE: standard error

Second, the relationship between the falls incidence and the trajectory of frailty progression was parameterised. As shown in Table 4, falls incidence is positively and significantly associated with frailty change, with the magnitude of association generally increasing by falls severity. An annual change in frailty score was assigned to individuals in the simulation model by halving the estimated frailty change from this regression. The feedback loop is thus established, with the now-higher frailty level increasing the risk of falling when the equation in Table 3 is re-applied in the next cycle. Figure A2 in the Supplementary Material illustrates the relation between severity of falls and change in frailty.

**Table 4:** Linear regression for two-year change in frailty

*Dependent variable: Change in frailty (range 0-100) between ELSA Waves 4 and 5* (*N* = *6,205)*		
Explanatory variables	Coefficient (SE)^1^	*P*-value
Constant	-5.460 (0.696)	< 0.001
Age in W4	0.134 (0.010)	< 0.001
SES (ref: Most privileged quartile)		
*2nd quartile*	0.089 (0.215)	0.680
*3rd quartile*	0.011 (0.184)	0.951
*Most deprived quartile*	0.701 (0.219)	0.001
Falls incidence between W4 and W5 (ref: No fall incidence)		
*Single non-MA fall*	0.684 (0.227)	0.003
*Recurrent non-MA falls*	2.329 (0.261)	< 0.001
*Single MA fall*	1.648 (0.350)	< 0.001
*Recurrent falls with MA*	3.870 (0.412)	< 0.001
Frailty in W4 (0-100)	-0.198 (0.010)	< 0.001
High physical activity in W4	-0.730 (0.192)	< 0.001
Cognitive impairment in W4	0.620 (0.187)	0.001
Social care receipt in W4	2.643 (0.589)	< 0.001
Informal care receipt in W4	1.612 (0.202)	< 0.001

^1^ Coefficient greater than zero implies the explanatory variable increased the odds of the dependent variable relative to its reference level, and vice versa.

Abbreviation: ELSA: English Longitudinal Study of Ageing; MA fall: fall requiring medical attention; Ref: reference; SE: standard error; SES: socioeconomic status; W4: ELSA Wave 4; W5: ELSA Wave 5

The conceptual association B concerning the secondary effects of falls is also relevant for this challenge. In addition to the *acute* QALY loss and care costs, the model should capture the longer-term impact on *comorbidity* health status and costs. These were parameterised as follows.

First, the longitudinal association between frailty change and EQ-5D-3 L health utility was estimated as shown in Table 5. How EQ-5D-3 L values were derived from ELSA is detailed elsewhere (see Appendix B of [33]). Based upon the regression reported in Table 5, falls affect the trajectory of EQ-5D-3 L in two ways: (i) directly via its association with EQ-5D-3 L change; and (ii) indirectly via the change in frailty. It should be noted that the falls incidence in ELSA could have occurred up to two years (i.e., the survey interval) prior to the EQ-5 L-3 L measurement. Hence, the direct association of (i) corresponds to the *non-acute* effect of falls, and the *acute* effect of falls on health utility is parameterised separately. This non-acute effect is minimal, with only one fall type being significantly associated with EQ-5D-3 L change (see Table A2 in the Supplementary Material which shows a stronger association when frailty change is removed as a covariate). The association of (ii) captures the indirect effect of falls on comorbidity health status.

**Table 5:** Linear regression for change in EQ-5D-3 L

*Dependent variable: Change in EQ-5D-3 L between ELSA Waves 4 and 5 (N = 6,205)*		
Explanatory variables	Coefficient (SE)	*P*-value
Constant	0.500 (0.025)	< 0.001
Age W4	0.002 (0.0003)	< 0.001
Female	-0.019 (0.005)	< 0.001
SES (ref: Most privileged quartile)		
*2nd quartile*	-0.019 (0.007)	0.008
*3rd quartile*	-0.009 (0.006)	0.162
*Most deprived quartile*	-0.023 (0.007)	0.002
Falls incidence W5 (ref: No fall incidence)		
*Single non-MA fall*	-0.013 (0.008)	0.081
*Recurrent non-MA falls*	-0.040 (0.009)	< 0.001
*Single MA fall*	-0.022 (0.012)	0.056
*Recurrent falls with MA*	-0.0001 (0.014)	0.943
Frailty W4 (0-100)	-0.010 (0.0004)	< 0.001
Change in frailty^1^	-0.014 (0.0004)	< 0.001
Abnormal gait/balance W4	-0.017 (0.007)	0.016
EQ-5D W4	-0.739 (0.034)	< 0.001
EQ-5D^2 W4	0.136 (0.029)	< 0.001

^1^ Two-year change in frailty between ELSA W4 and W5.

Abbreviation: ELSA: English Longitudinal Study of Ageing; MA fall: fall requiring medical attention; Ref: reference; SE: standard error; SES: socioeconomic status; W4: ELSA Wave 4; W5: ELSA Wave 5

**Fig. 3:**
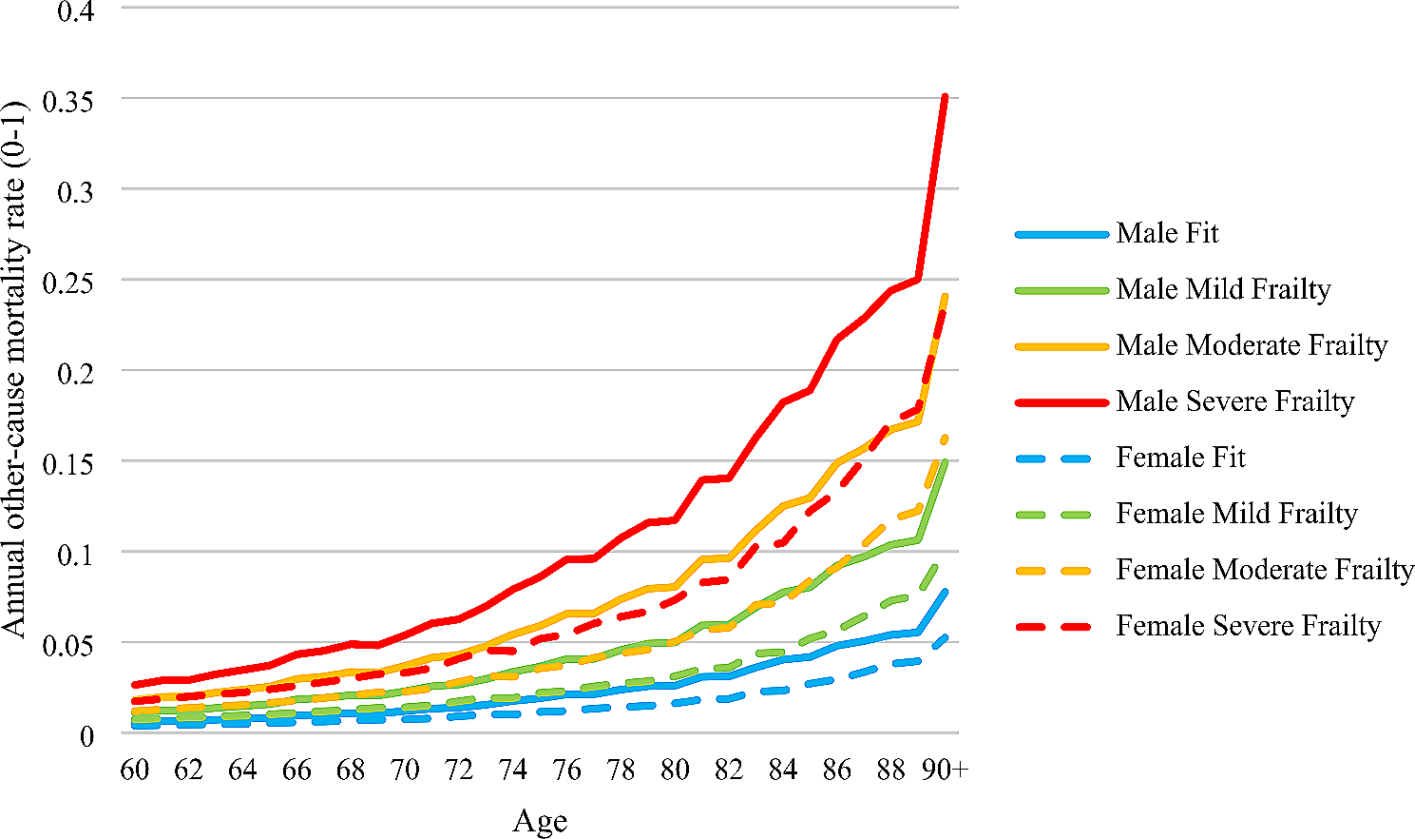
Annual other-cause mortality rate in community (range 0–1) by age, sex and frailty category

Second, the relationship between frailty and mortality risk was parameterised. Figure 3 shows the annual other-cause mortality rates for community-living adults aged 60 + by age, sex, and frailty category. Fatal falls comprised only small proportions of all-cause mortality and hence are not reported here: 0.76% of all deaths in men aged 50–69; 0.45% in women aged 50–69; 1.09% in men aged 70+; and 0.96% in women aged 70+ [49]. See Appendix B of [33] for details on how fall-related and other-cause mortality rates were estimated. Higher frailty change owing to falls thus induces higher mortality rates for causes other than fatal falls.

Third, the relationship between frailty and comorbidity care costs was similarly parameterised. Table A3 in the Supplementary Material shows the primary and secondary healthcare costs of comorbidities by frailty category, derived by subtracting the direct/acute fall-related costs from the all-cause costs. Therefore, increased frailty owing to falls induces higher comorbidity healthcare costs. The model also links frailty to costs of district nursing, short-term social care, and long-term care (privately and/or publicly funded) such that the frailty dynamic impacts these costs (see Appendix B of [33]).

Another way in which frailty shapes the long-term effects of a shock is to influence the access to interventions (conceptual association C). Tables A4 and A5 in the Supplementary Material show the logistic regressions estimating the likelihoods of accessing the GP (i.e., the proactive pathway) and demanding group exercise (i.e., the self-referred pathway), respectively. Frailty change is positively associated with both events, implying that fallers who experience higher rates of frailty change are more likely to access the proactive and/or self-referred pathways. The two pathways therefore dampen the falls-frailty feedback loop by potentially reducing the falls risks of frailer individuals.

### Incorporating a wide range of societal outcomes

The conceptual association most central to this challenge is B. The parameterisation of the various societal outcomes in the model proceeded similarly to that of the EQ-5D-3 L described above. Thus, Table 6 shows the results of logistic regression estimating the likelihood of engaging in regular (weekly or more) unpaid work, the prevalence of which (28.0%) was higher than that of paid employment (17.4%) in ELSA Waves 4–5. Table A6 in the Supplementary Material shows the results for the likelihood of being in paid employment. Both likelihoods were significantly and negatively associated with the level and change in frailty but not with falls incidence or history. The regressions therefore capture the indirect effect of falls on paid and unpaid contributions of older persons via frailty. The two contributions were valued using the human capital approach and the opportunity cost approach, respectively, as detailed elsewhere (Appendix B) [33]. Over the 40-year horizon, the discounted monetary value of the indirect productivity gain from RC relative to UC amounted to around £39 million [33].

**Table 6:** Logistic regression for engaging in regular unpaid work

*Dependent variable: Unpaid work* ^1^ *in Wave 5 (N = 6,205)*		
Explanatory variables	Coefficient (SE)^2^	*P*-value
Constant	-12.856 (2.951)	< 0.001
Age W4	0.331 (0.083)	< 0.001
Age^2 W4	-0.002 (0.0006)	< 0.001
Female	0.313 (0.065)	< 0.001
SES (ref: Most privileged quartile)		
*2nd quartile*	-0.266 (0.095)	0.005
*3rd quartile*	-0.233 (0.080)	0.004
*Most deprived quartile*	-0.236 (0.098)	0.016
Frailty W4 (0-100)	-0.013 (0.005)	0.010
Change in frailty^3^	-0.012 (0.006)	0.039
Cognitive impairment W4	-0.379 (0.091)	< 0.001
Abnormal gait/balance W4	-0.299 (0.097)	0.002
Unpaid work^1^ W4	1.944 (0.065)	< 0.001

^1^ ELSA W4-5 contained information on the frequency of ‘formal’ volunteering activities (i.e., as part of a volunteering organisation) in the past 12 months: at least once a week; less than once a week; and one-off. Similar frequency data was reported for provision of unpaid help (i.e., volunteering on a less formal basis), including informal caregiving for sick persons, childcare, and helping people with daily activities such as cooking, cleaning, and transporting. Together, they constituted unpaid work performed by older persons. A binary variable was created to indicate weekly or more regular unpaid work.

^2^ Coefficient greater than zero implies the explanatory variable increased the odds of the dependent variable relative to its reference level, and vice versa.

^3^ Two-year change in frailty between ELSA W4 and W5.

Abbreviation: ELSA: English Longitudinal Study of Ageing; MA fall: fall requiring medical attention; Ref: reference; SE: standard error; SES: socioeconomic status; W4: ELSA Wave 4; W5: ELSA Wave 5

Likewise, Table 7 shows the results of logistic regression estimating the likelihood of receiving care purchased OOP. The receipt was valued using the average hourly cost of private care and the information in ELSA on the weekly frequency of care visit which varied by frailty category and SES quartile (see Table A7 in the Supplementary Material). The significant positive associations between the likelihood and the level and change in frailty capture the indirect effect of falls on OOP care expenditure via frailty progression. Over the 40-year horizon, the discounted monetary value of RC’s impact on reducing the OOP care receipt relative to UC amounted to around £45 million [33].

**Table 7:** Logistic regression for out-of-pocket care receipt

*Dependent variable: OOP care receipt* ^1^ *in Wave 5 (N = 6,205)*		
Explanatory variables	Coefficient (SE)^2^	*P*-value
Constant	-10.011 (0.763)	< 0.001
Age W4	0.051 (0.010)	< 0.001
Female	0.712 (0.172)	< 0.001
SES (ref: Most privileged quartile)		
*2nd quartile*	-0.485 (0.227)	0.033
*3rd quartile*	-0.698 (0.193)	< 0.001
*Most deprived quartile*	-1.117 (0.247)	< 0.001
Frailty W4 (0-100)	0.174 (0.027)	< 0.001
Frailty^2 W4	-0.002 (0.0005)	< 0.001
Change in frailty^3^	0.063 (0.010)	< 0.001
High physical activity W4	-0.954 (0.435)	0.028
Fear of falling W4	0.659 (0.197)	0.001
OOP care receipt^1^ W4	1.851 (0.193)	< 0.001
Informal care receipt W4	-0.579 (0.181)	0.001

^1^ ELSA W4-5 contained information on the receipt of any privately paid help for activities of daily living.

^2^ Coefficient greater than zero implies the explanatory variable increased the odds of the dependent variable relative to its reference level, and vice versa.

^3^ Two-year change in frailty between ELSA W4 and W5.

Abbreviation: ELSA: English Longitudinal Study of Ageing; MA fall: fall requiring medical attention; Ref: reference; SE: standard error; SES: socioeconomic status; W4: ELSA Wave 4; W5: ELSA Wave 5; OOP: out-of-pocket

Table A8 in the Supplementary Material shows the results of logistic regression estimating the likelihood of receiving informal care. The significant positive associations between the likelihood and the level and change in frailty capture the indirect effect of falls on informal care need via frailty progression. The receipt was valued using the proxy goods approach, assuming that in the absence of informal care, individuals would purchase OOP care as a direct substitute [33]. ELSA information on whether an individual required informal care for single versus multiple activities of daily living was used as a measure of care intensity, and a separate logistic regression was estimated for requiring care for multiple activities [33]. Over the 40-year horizon, the discounted monetary value of RC’s impact on reducing informal care receipt relative to UC amounted to around £139 million [33].

### Accounting for heterogeneity

This section demonstrates how a measure of frailty can capture the heterogeneity within the demographic groups defined by age and sex. Table 8 shows the average values of key model health and cost outcomes by frailty category for men aged 60–69 years, serving here as an example of a demographic group. The outcome variations are clear, with the average values for the whole demographic group masking visible gradients to the outcomes across the frailty categories.

**Table 8:** Model health and cost outcomes by frailty category for men aged 60–69 years

Frailty category	Outcome mean (SE)^1^			
	EQ-5D-3 L index	QALY^2^	Annual fall-related primary and secondary care cost	Annual all-cause public sector care cost^2,3^
Fit	0.844 (0.0003)	2.867 (0.0100)	£30.79 (3.57)	£6,435 (22.62)
Mild	0.698 (0.0007)	2.712 (0.0091)	£98.14 (8.64)	£9,074 (32.05)
Moderate	0.443 (0.0017)	1.515 (0.0167)	£349.02 (49.04)	£12,574 (160.04)
Severe	0.170 (0.0066)	0.423 (0.0267)	£170.57 (81.97)	£17,248 (564.78)
All	0.759 (0.0007)	2.712 (0.0069)	£76.12 (4.84)	£7,847 (23.04)

^1^ All outcomes were measured at the end of the fifth model cycle under the recommended care (RC) scenario. The columns for EQ-5D-3 L index, fall-related care cost and all-cause care cost describe the outcomes during the fifth annual cycle.

^2^ Accumulated from the first to the fifth model cycle for individuals remaining in the community at the fifth model cycle.

^3^ Includes costs of fall-related primary and secondary healthcare, comorbidity primary and secondary healthcare, cost of dying, district nursing, short-term social care, and all-cause long-term care.

Abbreviation: QALY: quality-adjusted life year; SE: standard error.

**Fig. 4:**
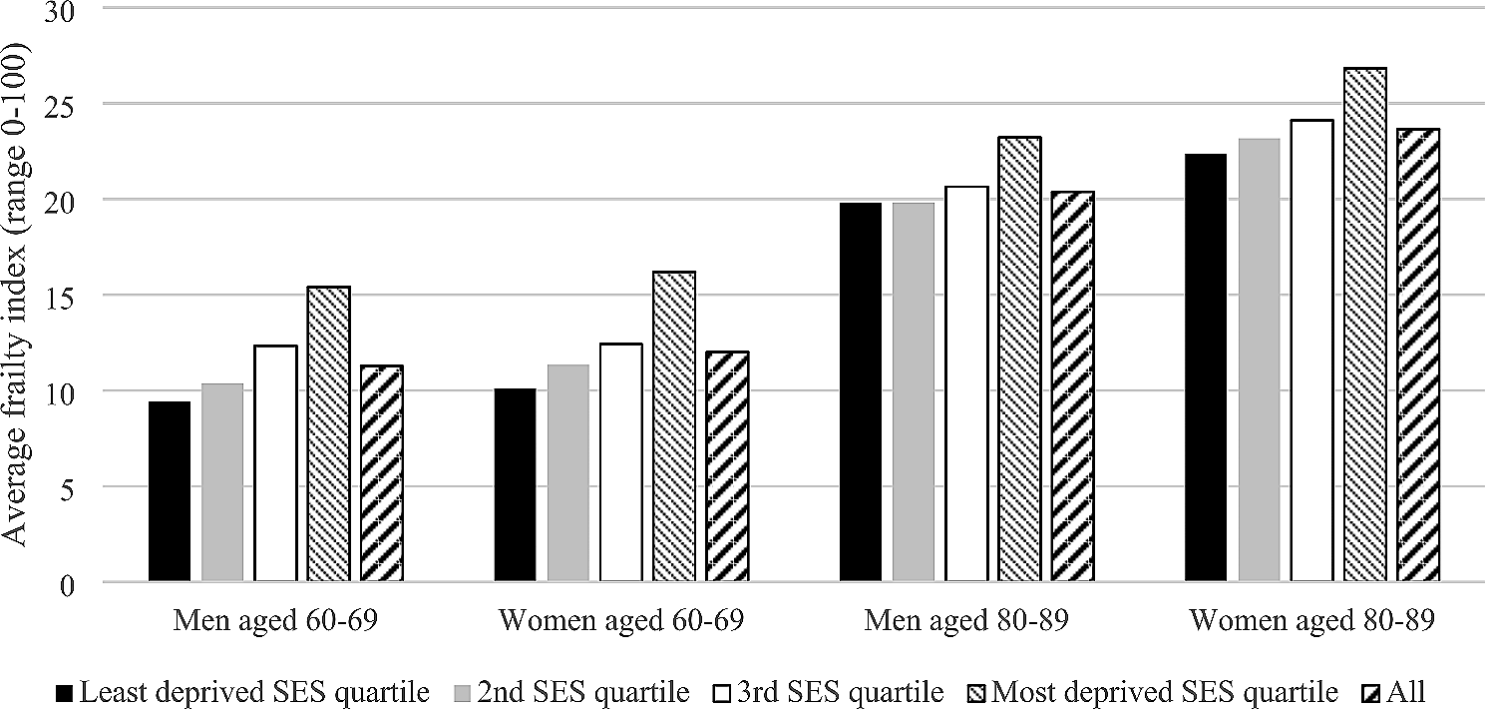
Average frailty index by demographic group and socioeconomic status quartile, in the fifth model cycle under recommended care. Abbreviation: SES: socioeconomic status

Finally, Fig. 4 shows the heterogeneity in the frailty level itself across the SES quartiles, with more socially deprived subgroups having higher frailty levels within each of the four demographic groups. Importantly, this heterogeneity would have equity implications if the inequalities in frailty-associated outcomes (i.e., all outcomes discussed above) across the SES quartiles are deemed unfair. The independent association between SES quartile and frailty change in Table 4, whereby the most deprived quartile experienced significantly higher rate of change, would also increase the inequalities over time. Overall, the conceptual associations A and B, parameterised as above, magnify the equity implications, while association C potentially mitigates it.

## Discussion

This article highlighted three challenges for the economic evaluation of geriatric interventions– (1) accounting for indirect, long-term impacts of geriatric shocks, (2) incorporating a wide range of societal outcomes, and (3) accounting for heterogeneity– and proposed a frailty-centred conceptual structure to address them. The structure encompassed three key associations involving frailty, and the structural validity of the final quantitative model would depend on identifying appropriate data and techniques to parameterise these associations. The recently developed DIS of community-based falls prevention [33] was presented as a case study, with the key component steps in its parameterisation being discussed. The level and change of the multivariate frailty index were shown to be associated with diverse model outcomes (e.g., EQ-5D-3 L change, unpaid work contribution, out-of-pocket care expenditure) and processes (e.g., GP access rate), such that frailty mediated the long-term effects of falls on health and non-health outcomes and explained outcome variations within demographic groups.

The key advantages of such frailty-based modelling become clear when it is compared to the methodological approaches of previous falls prevention models, 46 of which were identified and evaluated in a recent systematic review [50, 51]. First, of 17 previous models which had time horizons longer than five years, few incorporated time-varying risk factors for falls other than age and falls history [51]. Yet as is apparent in the literature [37, 52, 53] and in Table 3, falls have a multivariate risk profile encompassing more factors than age and falls history alone. A measure of frailty can capture the cumulative impact of the interactions between falls risk factors and serve as a summary indicator of the multivariate risk. Its complex dynamic interaction with falls and other factors (as parameterised in Table 4) can likewise capture the diversity of falls risk trajectories within any group defined by age and falls history.

Second, previous models relied on simplistic assumptions for characterising the long-term transitions in health utility and care costs following a severe fall [51]. Of the 17 models with time horizons longer than five years, only one allowed health utilities to vary by factors other than falls, age, sex, and ethnicity, specifically by binary indicator of functional dependency and long-term care admission status [54]. By contrast, incorporating the continuous frailty index (and further covariates) in Table 5 allowed the parameterisation of EQ-5D-3 L transition at an individual-level granularity. There was a similar lack of previous attempts at parameterising the long-term trajectory of comorbidity care costs: only nine models incorporated them at all, eight of which stratified them only by age, sex, ethnicity and/or falls [51]. As noted by Drummond and colleagues (p. 230-1) [16], there is a strong rationale for incorporating such comorbidity care costs: if evaluations of interventions assign all the credit for life extension using a generic measure of health gain, then it makes sense to assign all costs. Frailty modelling enables precisely this, i.e., to capture both the direct and indirect effects of a given shock on all cost outcomes.

Another prevalent limitation of previous falls prevention models has been the haphazard incorporation of non-health outcomes accrued outside the healthcare system [51]. Of the 18 models conducting evaluation from the societal perspective, four included OOP care expenditure, two informal caregiving cost, and only one productivity gain; others only incorporated societal intervention costs (e.g., time opportunity cost of participating in an intervention) [51]. By contrast, this article has shown how when the associations between frailty and various non-health outcomes are identified, then the economic model can incorporate the indirect effect of a given shock on these outcomes via frailty. Finally, few previous models accounted for heterogeneity by factors other than age, sex, and individual diseases (e.g., osteoporosis) [51]. This precludes not only the conduct of comprehensive subgroup analyses but also the evaluation of intervention targeting based on frailty. Such targeting may be necessary under capacity constraints, as illustrated by a local scheme in Sheffield, UK, wherein falls prevention access was targeted at those who are moderately frail according to the electronic frailty index [36]. Modelling of frailty, whether as a categorical or continuous variable, enables such evaluations.

A key challenge to (geriatric or non-geriatric) public health economic evaluation– which was not explicitly considered in this article– is addressing the issues of equity [55–57]. It is nevertheless clear that handling the three challenges considered in this article is a highly relevant step. Most explicitly, the challenge of accounting for heterogeneity involved identifying the social gradients to frailty and to frailty-associated model outcomes. Frailty thus mediates inequalities in various key outcomes within demographic groups. If such social inequalities are deemed unfair, the heterogeneity provides the platform for evaluating the equity-efficiency trade-off of interventions. Specifically, techniques such as distributional cost-effectiveness analysis (DCEA) can be used as applied within the current DIS model [33].

Addressing the other two challenges likewise have equity implications. The inclusion of non-health outcomes likely exacerbates social inequity [33]. This is apparent from the findings in Tables 6 and 7, for example, that the most privileged SES quartile is engaged in significantly higher unpaid work level and receives significantly higher OOP care. Benefits of interventions that promote unpaid work and reduce OOP care would likely accrue to this quartile disproportionately. Accounting for indirect, long-term effect of a shock likely has a more ambiguous impact. The socially advantaged groups with longer life expectancies likely benefit more from an intervention that improves their comorbidity health outcomes and/or reduce their comorbidity care costs. By contrast, incorporating the shock-frailty feedback loop may grant greater intervention benefit to the socially deprived, since an intervention that can successfully dampen the loop would benefit more those who are frailer at the outset. Indeed, a counterfactual scenario that removed the feedback loop in the current DIS model made RC no longer equity-improving relative to UC [33]. Overall, addressing the three challenges enables a nuanced, joint evaluation of efficiency and equity of public health interventions, and the model development should involve stakeholder consultations on the vulnerable subgroups warranting priority [10, 19, 58].

It should be noted that the methods used to parameterise the current DIS model are not the unique, let alone the optimal, means of quantifying and operationalising the conceptual structure in Fig. 1. Alternative data sources and statistical methods should be used if they can improve several aspects of the parameterisation. For example, estimates of the individual-level associations between frailty level (rather than category as in Table A3 and Fig. 3) and comorbidity care costs and mortality risk would increase the granularity of the indirect effects of shocks via frailty progression. Non-linear regressions could capture the drop in paid employment rate after age 65. Statistical methods for causal inference could also be used, particularly when estimating the longitudinal trajectories of frailty. The ELSA data moreover carried several limitations, such as the sample attrition between Waves and the recall bias in the measurement of falls. This case study also used ELSA Waves 4 and 5, rather than the more recent Waves, due to the greater availability of fall-related variables in Wave 4 [33]. It should nonetheless be noted that model parameterisation will always be constrained by the available data and modelling techniques [10]. No dataset will perfectly suit the modelling need, and in this case, ELSA had strengths relevant to the project (e.g., having data on productivity and informal care receipt). That ELSA is publicly available also means that the methods here can be easily replicated. Likewise, the analyst should ensure that parameter estimates obtained from more complex statistical methods are tractable for coding within the modelling software (in this case Simul8).

Other study caveats can be noted. First, the three challenges discussed here do not exhaust the range of contributions made by frailty to economic modelling. Indeed, the current DIS model has explored further roles, including: (a) a lower baseline frailty of the target population summating the impact of successful earlier-life preventions and affecting the cost-effectiveness of falls prevention [33]; and (b) targeting interventions based on frailty under capacity constraints, with this being compared to other targeting methods [42]. Second, how frailty might influence intervention efficacy was not discussed (unlike its influence on intervention access under association C), even though evidence suggests that efficacy can vary by frailty [17, 18]. Third, the conceptual structure in Fig. 1 focused on how interventions affect frailty *indirectly* via reducing the geriatric shock, but some interventions might aim to reduce frailty *directly* [59]. Figure 1 also conceptualised interventions indirectly affecting outcomes such as productivity via frailty. However, some interventions might seek to improve such outcomes *given* a frailty level. For example, NICE recommends that the paid and unpaid contributions of older persons be promoted to reduce social isolation, without this necessarily reducing the underlying frailty [60].

The conceptual structure and parameterisation methods used in this case study are relevant for other geriatric and non-geriatric fields. The bidirectional feedback loop between frailty and falls is likely present between frailty and other geriatric syndromes including dementia [61–63]. The need for models to incorporate a wide range of societal, non-health outcomes has likewise been highlighted in other disease areas [15, 57, 64–67]. Models of earlier-life interventions such as diabetes prevention (e.g., [68]) could incorporate the frailty-based associations at the later life-course stages of the modelled population to capture the relevant dynamics.

Frailty could moreover play a vital role in intervention design: frailty or similar multivariate indices calculated from electronic primary care records could stratify individuals by risk of adverse events and prioritise intervention access to those with the greatest need [25, 36, 69]. Development of a simple-to-use online risk calculator, such as that for cardiovascular risk [70], would greatly aid the implementation. Under constrained intervention capacity, the use of screening tools with low sensitivity and specificity may result in referral rates that outstrip the intervention capacity. For instance, the current model estimates that seven full-time falls clinics would be required to fully implement RC based on NICE guideline [33]. In this scenario, an additional frailty-based targeting appears apt. Furthermore, a simulation model that can characterise capacity constraints, such as the current DIS model, will play a vital role in evaluating the cost-effectiveness and equity of different targeting strategies.

## Conclusion

This article presents the details of a case study of falls prevention economic modelling which extensively used a multivariate frailty index to generate the dynamics and outcomes relevant to decision-making in geriatric health. It was demonstrated specifically how frailty modelling can contribute to accounting for indirect, long-term effects of geriatric shocks, incorporating a wide range of societal outcomes, and accounting for heterogeneity. The conceptual structure of frailty’s multi-faceted contribution is applicable to a broad range of geriatric and non-geriatric conditions. The conceptual associations should be parameterised using appropriate data and statistical methods to develop structurally valid and credible economic models of geriatric interventions.

### Electronic supplementary material

Below is the link to the electronic supplementary material.

**Supplementary Material 1**: The Supplementary Material contains Tables A1-A8 and Figures A1-A2

## Data Availability

The documents informing the model conceptualisation, the Simul8 model file, and the model outputs generated by the current study are available from the corresponding author on reasonable request. The English Longitudinal Study of Ageing (ELSA), the main source of data for model parameterisation, can be accessed from the UK Data Service: https://beta.ukdataservice.ac.uk/datacatalogue/series/series?id=200011.

## Abbreviations

Aged 60+: Aged 60 and over
DCEA: Distributional cost-effectiveness analysis
DIS: Discrete individual simulation
ELSA: English Longitudinal Study of Ageing
NICE: National Institute for Health and Care Excellence
OOP: Out-of-pocket
QALY: Quality-adjusted life year
RC: Recommended care
SD: Standard deviation
SES: Socioeconomic status
UC: Usual care
