# Supplementary material for: Incorporating frailty to address the key challenges to geriatric economic evaluation
Source: BMC Geriatr. 2024 Feb 14;24:155. doi: 10.1186/s12877-024-04752-5 (PMC10868084; doi:10.1186/s12877-024-04752-5)
Supplement: Supplementary file 1 — Supplementary Material 1: The Supplementary Material contains Tables A1-A8 and Figures A1-A2 [file 12877_2024_4752_MOESM1_ESM.docx]

# Incorporating frailty to address the key challenges to geriatric economic evaluation

**Supplementary Material**

**Running title:** Frailty modelling for geriatric economic evaluation

**Keywords:** frailty; falls prevention; economic model; long-term outcomes; societal outcomes; equity

**Authors:**

Dr Joseph Kwon,^1*^ [joseph.kwon@phc.ox.ac.uk](mailto:joseph.kwon@phc.ox.ac.uk); ORCID 0000-0002-2860-7280

Dr Hazel Squires,^2^ [h.squires@sheffield.ac.uk](mailto:h.squires@sheffield.ac.uk); ORCID 0000-0001-8467-0471

Professor Tracey Young,^2^ [t.a.young@sheffield.ac.uk](mailto:t.a.young@sheffield.ac.uk); ORCID 0000-0002-0754-7223

^1^ Nuffield Department of Primary Care Health Sciences, University of Oxford, Radcliffe Primary Care Building, Radcliffe Observatory Quarter, Woodstock Road, Oxford, England, OX2 6GG

^2^ School of Health and Related Research, University of Sheffield, Regent Court (ScHARR), 30 Regent Street, Sheffield, England, S1 4DA

* Corresponding author

**Competing interest:** The authors declare that they have no competing interests.

**Funding:** This research was funded in whole, or in part, by the Wellcome Trust [108903/B/15/Z]. For the purpose of Open Access, the author has applied a CC BY public copyright licence to any Author Accepted Manuscript version arising from this submission.

# Multivariate frailty index

The literature recommends that a multivariate frailty index should: (i) incorporate at least 30 deficit items and that each item should be an adverse health outcome associated with age; (ii) not saturated at early old age; (iii) have prevalence greater than 1%; and (iv) have less than 5% missing values in study sample [1]. Accordingly, each deficit item was checked for: (i) increasing prevalence with age; (ii) less than 100% prevalence at age 65 – a criterion used for eFI [2]; and (iii) greater than 1% prevalence across the whole population aged 60 and over – which excluded Alzheimer’s disease or dementia, Parkinson’s disease, and schizophrenia or psychosis. Six variables – gait speed (time to walk 8 feet), self-reported balance problem severity, grip strength (weight in kg lifted with dominant hand), leg strength (time to complete five chair rises), weight and body mass index category – had more than 5% missing data. Data for these variables were imputed using multivariate single imputation.

Table A1 shows the mean and standard deviation parameters for lognormal distributions of multivariate frailty scores (range 0-100) assigned to simulated individuals by five-year age group, sex, social deprivation quartile, and baseline falls history type.

| **Table A1** Mean and standard deviation of lognormally distributed frailty index (range 0-100) by five-year age group, sex, social deprivation quartile and falls history type. | | | | | | |
| --- | --- | --- | --- | --- | --- | --- |
| ***Male in 1^st^ social quartile (most privileged)*** | | | | | | |
| MS1Age1FH0  Mean: 6.40  SD: 5.41 | MS1Age2FH0  Mean: 7.33  SD: 6.22 | MS1Age3FH0  Mean: 9.10  SD: 7.30 | MS1Age4FH0  Mean: 11.55  SD: 7.28 | MS1Age5FH0  Mean: 15.15  SD: 9.24 | MS1Age6FH0  Mean: 17.99  SD: 9.69 | MS1Age7FH0  Mean: 24.13  SD: 11.24 |
| MS1Age1FH1  Mean: 6.59  SD: 5.76 | MS1Age2FH1  Mean: 8.86  SD: 6.54 | MS1Age3FH1  Mean: 11.47  SD: 8.00 | MS1Age4FH1  Mean: 11.83  SD: 8.31 | MS1Age5FH1  Mean: 18.51  SD: 10.16 | MS1Age6FH1  Mean: 15.54  SD: 7.31 | MS1Age7FH1  Mean: 16.99  SD: 12.00 |
| MS1Age1FH2  Mean: 12.73  SD: 11.98 | MS1Age2FH2  Mean: 13.60  SD: 8.72 | MS1Age3FH2  Mean: 19.96  SD: 12.81 | MS1Age4FH2  Mean: 19.80  SD: 11.26 | MS1Age5FH2  Mean: 24.48  SD: 10.87 | MS1Age6FH2  Mean: 27.31  SD: 13.07 | MS1Age7FH2  Mean: 22.55  SD: 15.10 |
| MS1Age1FH3  Mean: 17.31  SD: 13.00 | MS1Age2FH3  Mean: 8.38  SD: 4.17 | MS1Age3FH3  Mean: 10.15  SD: 5.67 | MS1Age4FH3  Mean: 15.72  SD: 8.14 | MS1Age5FH3  Mean: 19.87  SD: 13.36 | MS1Age6FH3  Mean: 12.50  SD: 4.00 | MS1Age7FH3  Mean: 15.18  SD: 10.41 |
| MS1Age1FH4  Mean: 9.23  SD: 3.70 | MS1Age2FH4  Mean: 14.23  SD: 9.34 | MS1Age3FH4  Mean: 18.34  SD: 14.15 | MS1Age4FH4  Mean: 25.00  SD: 9.25 | MS1Age5FH4  Mean: 25.00  SD: 13.32 | MS1Age6FH4  Mean: 18.80  SD: 5.99 | MS1Age7FH4  Mean: 24.04  SD: 4.08 |
| ***Male in 2^nd^ social quartile*** | | | | | | |
| MS2Age1FH0  Mean: 6.40  SD: 5.41 | MS2Age2FH0  Mean: 7.01  SD: 5.78 | MS2Age3FH0  Mean: 8.53  SD: 6.69 | MS2Age4FH0  Mean: 10.51  SD: 8.19 | MS2Age5FH0  Mean: 11.89  SD: 8.45 | MS2Age6FH0  Mean: 15.26  SD: 9.76 | MS2Age7FH0  Mean: 18.09  SD: 11.35 |
| MS2Age1FH1  Mean: 6.59  SD: 5.76 | MS2Age2FH1  Mean: 10.19  SD: 9.31 | MS2Age3FH1  Mean: 11.41  SD: 8.37 | MS2Age4FH1  Mean: 8.72  SD: 7.33 | MS2Age5FH1  Mean: 16.97  SD: 9.13 | MS2Age6FH1  Mean: 13.94  SD: 10.06 | MS2Age7FH1  Mean: 12.26  SD: 7.04 |
| MS2Age1FH2  Mean: 12.73  SD: 11.98 | MS2Age2FH2  Mean: 18.09  SD: 12.57 | MS2Age3FH2  Mean: 16.09  SD: 12.76 | MS2Age4FH2  Mean: 20.19  SD: 14.05 | MS2Age5FH2  Mean: 19.97  SD: 15.40 | MS2Age6FH2  Mean: 24.83  SD: 9.28 | MS2Age7FH2  Mean: 31.92  SD: 5.70 |
| MS2Age1FH3  Mean: 17.31  SD: 13.00 | MS2Age2FH3  Mean: 3.85  SD: 2.72 | MS2Age3FH3  Mean: 16.03  SD: 15.89 | MS2Age4FH3  Mean: 11.86  SD: 9.22 | MS2Age5FH3  Mean: 16.08  SD: 11.19 | MS2Age6FH3  Mean: 23.46  SD: 16.17 | MS2Age7FH3  Mean: 18.41  SD: 9.54 |
| MS2Age1FH4  Mean: 9.23  SD: 3.70 | MS2Age2FH4  Mean: 14.42  SD: 1.92 | MS2Age3FH4  Mean: 36.54  SD: 5.44 | MS2Age4FH4  Mean: 23.08  SD: 11.64 | MS2Age5FH4  Mean: 36.54  SD: 11.04 | MS2Age6FH4  Mean: 31.92  SD: 6.46 | MS2Age7FH4  Mean: 26.68  SD: 8.69 |
| ***Male in 3^rd^ social quartile*** | | | | | | |
| MS3Age1FH0  Mean: 9.38  SD: 8.69 | MS3Age2FH0  Mean: 10.32  SD: 7.91 | MS3Age3FH0  Mean: 11.60  SD: 8.47 | MS3Age4FH0  Mean: 13.22  SD: 9.69 | MS3Age5FH0  Mean: 14.78  SD: 8.32 | MS3Age6FH0  Mean: 18.25  SD: 10.05 | MS3Age7FH0  Mean: 27.35  SD: 10.87 |
| MS3Age1FH1  Mean: 11.40  SD: 9.70 | MS3Age2FH1  Mean: 10.95  SD: 8.80 | MS3Age3FH1  Mean: 15.14  SD: 10.58 | MS3Age4FH1  Mean: 16.11  SD: 9.70 | MS3Age5FH1  Mean: 15.58  SD: 9.00 | MS3Age6FH1  Mean: 17.31  SD: 5.90 | MS3Age7FH1  Mean: 29.80  SD: 6.51 |
| MS3Age1FH2  Mean: 19.28  SD: 12.75 | MS3Age2FH2  Mean: 19.86  SD: 11.62 | MS3Age3FH2  Mean: 24.54  SD: 12.17 | MS3Age4FH2  Mean: 22.79  SD: 8.85 | MS3Age5FH2  Mean: 20.10  SD: 8.22 | MS3Age6FH2  Mean: 20.73  SD: 11.49 | MS3Age7FH2  Mean: 21.79  SD: 6.18 |
| MS3Age1FH3  Mean: 18.11  SD: 10.16 | MS3Age2FH3  Mean: 23.08  SD: 15.90 | MS3Age3FH3  Mean: 13.85  SD: 8.25 | MS3Age4FH3  Mean: 21.75  SD: 11.39 | MS3Age5FH3  Mean: 18.85  SD: 17.75 | MS3Age6FH3  Mean: 23.56  SD: 9.21 | MS3Age7FH3  Mean: 26.92  SD: 5.44 |
| MS3Age1FH4  Mean: 11.78  SD: 10.40 | MS3Age2FH4  Mean: 14.42  SD: 5.27 | MS3Age3FH4  Mean: 19.78  SD: 11.03 | MS3Age4FH4  Mean: 18.68  SD: 9.40 | MS3Age5FH4  Mean: 30.77  SD: 1.92 | MS3Age6FH4  Mean: 22.55  SD: 9.26 | MS3Age7FH4  Mean: 30.77  SD: 3.57 |
| ***Male in 4^th^ social quartile*** | | | | | | |
| MS4Age1FH0  Mean: 11.40  SD: 8.54 | MS4Age2FH0  Mean: 14.13  SD: 9.64 | MS4Age3FH0  Mean: 13.44  SD: 9.37 | MS4Age4FH0  Mean: 15.49  SD: 11.72 | MS4Age5FH0  Mean: 15.00  SD: 8.56 | MS4Age6FH0  Mean: 19.78  SD: 14.21 | MS4Age7FH0  Mean: 24.04  SD: 11.05 |
| MS4Age1FH1  Mean: 14.93  SD: 8.61 | MS4Age2FH1  Mean: 12.59  SD: 8.79 | MS4Age3FH1  Mean: 15.38  SD: 8.55 | MS4Age4FH1  Mean: 18.59  SD: 8.33 | MS4Age5FH1  Mean: 23.50  SD: 12.08 | MS4Age6FH1  Mean: 22.23  SD: 12.08 | MS4Age7FH1  Mean: 25.00  SD: 5.44 |
| MS4Age1FH2  Mean: 25.25  SD: 10.01 | MS4Age2FH2  Mean: 19.14  SD: 9.62 | MS4Age3FH2  Mean: 21.27  SD: 11.84 | MS4Age4FH2  Mean: 21.54  SD: 5.67 | MS4Age5FH2  Mean: 32.05  SD: 12.08 | MS4Age6FH2  Mean: 28.85  SD: 12.08 | MS4Age7FH2  Mean: 27.11  SD: 12.08 |
| MS4Age1FH3  Mean: 10.58  SD: 4.51 | MS4Age2FH3  Mean: 20.19  SD: 20.40 | MS4Age3FH3  Mean: 14.42  SD: 5.55 | MS4Age4FH3  Mean: 19.71  SD: 13.08 | MS4Age5FH3  Mean: 32.05  SD: 13.64 | MS4Age6FH3  Mean: 17.31  SD: 13.64 | MS4Age7FH3  Mean: 19.74  SD: 13.64 |
| MS4Age1FH4  Mean: 27.56  SD: 14.56 | MS4Age2FH4  Mean: 27.88  SD: 17.68 | MS4Age3FH4  Mean: 23.56  SD: 3.28 | MS4Age4FH4  Mean: 15.11  SD: 8.49 | MS4Age5FH4  Mean: 20.19  SD: 8.08 | MS4Age6FH4  Mean: 34.62  SD: 8.08 | MS4Age7FH4  Mean: 28.85  SD: 8.08 |
| ***Female in 1^st^ social quartile (most privileged)*** | | | | | | |
| FS1Age1FH0  Mean: 7.10  SD: 5.78 | FS1Age2FH0  Mean: 8.62  SD: 6.80 | FS1Age3FH0  Mean: 11.40  SD: 7.84 | FS1Age4FH0  Mean: 14.45  SD: 8.60 | FS1Age5FH0  Mean: 17.35  SD: 10.19 | FS1Age6FH0  Mean: 22.83  SD: 12.45 | FS1Age7FH0  Mean: 27.98  SD: 12.55 |
| FS1Age1FH1  Mean: 6.85  SD: 5.81 | FS1Age2FH1  Mean: 10.24  SD: 7.83 | FS1Age3FH1  Mean: 12.46  SD: 8.43 | FS1Age4FH1  Mean: 13.77  SD: 6.85 | FS1Age5FH1  Mean: 18.37  SD: 10.60 | FS1Age6FH1  Mean: 21.29  SD: 9.50 | FS1Age7FH1  Mean: 31.25  SD: 11.14 |
| FS1Age1FH2  Mean: 10.81  SD: 8.35 | FS1Age2FH2  Mean: 11.68  SD: 9.35 | FS1Age3FH2  Mean: 12.91  SD: 9.49 | FS1Age4FH2  Mean: 17.81  SD: 9.84 | FS1Age5FH2  Mean: 24.04  SD: 10.46 | FS1Age6FH2  Mean: 25.59  SD: 11.11 | FS1Age7FH2  Mean: 33.41  SD: 12.25 |
| FS1Age1FH3  Mean: 8.85  SD: 5.83 | FS1Age2FH3  Mean: 10.40  SD: 8.54 | FS1Age3FH3  Mean: 11.43  SD: 5.46 | FS1Age4FH3  Mean: 16.06  SD: 9.55 | FS1Age5FH3  Mean: 21.02  SD: 10.89 | FS1Age6FH3  Mean: 20.51  SD: 7.54 | FS1Age7FH3  Mean: 21.63  SD: 6.55 |
| FS1Age1FH4  Mean: 14.79  SD: 11.44 | FS1Age2FH4  Mean: 15.77  SD: 9.67 | FS1Age3FH4  Mean: 17.44  SD: 9.93 | FS1Age4FH4  Mean: 34.38  SD: 17.09 | FS1Age5FH4  Mean: 26.79  SD: 9.74 | FS1Age6FH4  Mean: 35.64  SD: 7.15 | FS1Age7FH4  Mean: 40.38  SD: 21.47 |
| ***Female in 2^nd^ social quartile*** | | | | | | |
| FS2Age1FH0  Mean: 7.79  SD: 5.62 | FS2Age2FH0  Mean: 9.85  SD: 8.60 | FS2Age3FH0  Mean: 11.60  SD: 7.99 | FS2Age4FH0  Mean: 16.44  SD: 10.23 | FS2Age5FH0  Mean: 17.22  SD: 9.23 | FS2Age6FH0  Mean: 20.49  SD: 11.29 | FS2Age7FH0  Mean: 28.85  SD: 8.76 |
| FS2Age1FH1  Mean: 8.82  SD: 5.50 | FS2Age2FH1  Mean: 12.43  SD: 9.28 | FS2Age3FH1  Mean: 13.18  SD: 9.39 | FS2Age4FH1  Mean: 17.31  SD: 9.52 | FS2Age5FH1  Mean: 18.22  SD: 11.63 | FS2Age6FH1  Mean: 21.43  SD: 11.65 | FS2Age7FH1  Mean: 25.38  SD: 7.50 |
| FS2Age1FH2  Mean: 17.08  SD: 11.11 | FS2Age2FH2  Mean: 14.18  SD: 13.09 | FS2Age3FH2  Mean: 18.79  SD: 11.04 | FS2Age4FH2  Mean: 23.87  SD: 16.08 | FS2Age5FH2  Mean: 21.03  SD: 9.97 | FS2Age6FH2  Mean: 25.27  SD: 8.34 | FS2Age7FH2  Mean: 29.67  SD: 13.82 |
| FS2Age1FH3  Mean: 10.21  SD: 11.60 | FS2Age2FH3  Mean: 11.15  SD: 10.00 | FS2Age3FH3  Mean: 11.78  SD: 6.29 | FS2Age4FH3  Mean: 17.19  SD: 8.44 | FS2Age5FH3  Mean: 20.55  SD: 13.52 | FS2Age6FH3  Mean: 21.35  SD: 11.52 | FS2Age7FH3  Mean: 27.56  SD: 7.77 |
| FS2Age1FH4  Mean: 19.23  SD: 14.99 | FS2Age2FH4  Mean: 17.12  SD: 8.62 | FS2Age3FH4  Mean: 6.41  SD: 4.33 | FS2Age4FH4  Mean: 28.85  SD: 14.50 | FS2Age5FH4  Mean: 27.50  SD: 11.68 | FS2Age6FH4  Mean: 28.85  SD: 19.04 | FS2Age7FH4  Mean: 30.13  SD: 10.94 |
| ***Female in 3^rd^ social quartile*** | | | | | | |
| FS3Age1FH0  Mean: 9.13  SD: 7.21 | FS3Age2FH0  Mean: 10.95  SD: 8.38 | FS3Age3FH0  Mean: 13.08  SD: 8.27 | FS3Age4FH0  Mean: 15.34  SD: 9.00 | FS3Age5FH0  Mean: 20.52  SD: 11.17 | FS3Age6FH0  Mean: 21.37  SD: 9.84 | FS3Age7FH0  Mean: 27.69  SD: 14.30 |
| FS3Age1FH1  Mean: 12.02  SD: 9.68 | FS3Age2FH1  Mean: 11.61  SD: 7.34 | FS3Age3FH1  Mean: 16.29  SD: 12.88 | FS3Age4FH1  Mean: 16.58  SD: 8.00 | FS3Age5FH1  Mean: 18.80  SD: 8.29 | FS3Age6FH1  Mean: 25.92  SD: 10.11 | FS3Age7FH1  Mean: 27.24  SD: 7.64 |
| FS3Age1FH2  Mean: 17.27  SD: 12.12 | FS3Age2FH2  Mean: 18.89  SD: 11.67 | FS3Age3FH2  Mean: 19.03  SD: 10.96 | FS3Age4FH2  Mean: 27.11  SD: 13.38 | FS3Age5FH2  Mean: 24.08  SD: 11.54 | FS3Age6FH2  Mean: 29.81  SD: 11.29 | FS3Age7FH2  Mean: 38.14  SD: 15.69 |
| FS3Age1FH3  Mean: 7.55  SD: 5.90 | FS3Age2FH3  Mean: 12.82  SD: 7.12 | FS3Age3FH3  Mean: 14.65  SD: 8.76 | FS3Age4FH3  Mean: 22.41  SD: 9.36 | FS3Age5FH3  Mean: 17.84  SD: 7.66 | FS3Age6FH3  Mean: 22.78  SD: 11.10 | FS3Age7FH3  Mean: 19.23  SD: 41.42 |
| FS3Age1FH4  Mean: 19.23  SD: 12.92 | FS3Age2FH4  Mean: 20.64  SD: 15.24 | FS3Age3FH4  Mean: 29.81  SD: 14.53 | FS3Age4FH4  Mean: 24.65  SD: 11.43 | FS3Age5FH4  Mean: 28.67  SD: 9.44 | FS3Age6FH4  Mean: 29.57  SD: 11.72 | FS3Age7FH4  Mean: 34.23  SD: 20.05 |
| ***Female in 4^th^ social quartile*** | | | | | | |
| FS4Age1FH0  Mean: 12.52  SD: 7.24 | FS4Age2FH0  Mean: 14.23  SD: 9.28 | FS4Age3FH0  Mean: 14.11  SD: 8.97 | FS4Age4FH0  Mean: 17.61  SD: 9.68 | FS4Age5FH0  Mean: 21.98  SD: 12.08 | FS4Age6FH0  Mean: 19.66  SD: 9.22 | FS4Age7FH0  Mean: 22.80  SD: 5.71 |
| FS4Age1FH1  Mean: 13.40  SD: 8.88 | FS4Age2FH1  Mean: 17.31  SD: 11.17 | FS4Age3FH1  Mean: 16.24  SD: 9.46 | FS4Age4FH1  Mean: 20.62  SD: 9.51 | FS4Age5FH1  Mean: 21.88  SD: 12.25 | FS4Age6FH1  Mean: 28.37  SD: 5.30 | FS4Age7FH1  Mean: 25.00  SD: 5.30 |
| FS4Age1FH2  Mean: 24.56  SD: 9.29 | FS4Age2FH2  Mean: 22.21  SD: 17.08 | FS4Age3FH2  Mean: 23.61  SD: 12.99 | FS4Age4FH2  Mean: 28.63  SD: 11.59 | FS4Age5FH2  Mean: 29.81  SD: 16.02 | FS4Age6FH2  Mean: 28.53  SD: 17.93 | FS4Age7FH2  Mean: 51.92  SD: 17.93 |
| FS4Age1FH3  Mean: 10.19  SD: 9.55 | FS4Age2FH3  Mean: 18.18  SD: 9.24 | FS4Age3FH3  Mean: 16.42  SD: 6.41 | FS4Age4FH3  Mean: 33.79  SD: 17.69 | FS4Age5FH3  Mean: 23.46  SD: 10.29 | FS4Age6FH3  Mean: 24.04  SD: 17.68 | FS4Age7FH3  Mean: 44.55  SD: 17.68 |
| FS4Age1FH4  Mean: 18.65  SD: 10.57 | FS4Age2FH4  Mean: 27.66  SD: 14.40 | FS4Age3FH4  Mean: 32.93  SD: 11.83 | FS4Age4FH4  Mean: 27.56  SD: 8.16 | FS4Age5FH4  Mean: 32.97  SD: 12.83 | FS4Age6FH4  Mean: 33.17  SD: 9.85 | FS4Age7FH4  Mean: 38.46  SD: 9.85 |
| **Subgroup abbreviations:** MS1Age1FH0 – male, 1^st^ social quartile (most privileged), 1^st^ age group (60-64) and falls history type 0 (no falls history); FS4Age5FH3 – female, 4^th^ social quartile (most deprived), 5^th^ age group (80-84) and falls history type 3 (single MA fall history); FH0 – no falls history; FH1 – single fall not requiring medical attention; FH2 – recurrent falls not requiring medical attention; FH3 – single fall requiring medical attention; FH4 – two or more falls with at least one fall requiring medical attention. | | | | | | |

# (1) Accounting for indirect, long-term effects of shock

**Figure A1** Risk of any fall by frailty category in fifth model cycle.

**Figure A2** Annual change in frailty score between fourth and fifth model cycle.

| **Table A2** Linear regression for change in EQ-5D-3L after removing frailty change as covariate. | | |
| --- | --- | --- |
| ***Dependent variable: Change in EQ-5D-3L between ELSA Waves 4 and 5 (N=6,205)*** | | |
| **Explanatory variables** | **Coefficient (SE)** | **P-value** |
| Constant | 0.584 (0.027) | <0.001 |
| Age W4 | 0.0004 (0.0004) | 0.316 |
| Female | -0.023 (0.005) | <0.001 |
| SES (ref: Most privileged quartile) |  |  |
| *2^nd^ quartile* | -0.020 (0.008) | 0.009 |
| *3^rd^ quartile* | -0.009 (0.007) | 0.193 |
| *Most deprived quartile* | -0.032 (0.008) | <0.001 |
| Falls incidence W5 (ref: No fall incidence) |  |  |
| *Single non-MA fall* | -0.021 (0.008) | 0.009 |
| *Recurrent non-MA falls* | -0.072 (0.009) | <0.001 |
| *Single MA fall* | -0.046 (0.013) | <0.001 |
| *Recurrent falls with MA* | -0.052 (0.015) | <0.001 |
| Frailty W4 (0-100) | -0.007 (0.0004) | <0.001 |
| Abnormal gait/balance W4 | -0.022 (0.008) | 0.004 |
| EQ-5D W4 | -0.782 (0.037) | <0.001 |
| EQ-5D^2 W4 | 0.193 (0.031) | <0.001 |
| **Abbreviation:** ELSA: English Longitudinal Study of Ageing; MA fall: fall requiring medical attention; Ref: reference; SE: standard error; SES: socioeconomic status; W4: ELSA Wave 4; W5: ELSA Wave 5 | | |

| **Table A3** Annual all-cause and comorbidity primary and secondary healthcare costs by frailty category. | | | |
| --- | --- | --- | --- |
| **Frailty category** | **Annual all-cause primary and secondary healthcare cost** | | **Annual comorbidity primary and secondary healthcare cost** |
|  | **2013/14 £^1^** | **2021/22 £^2^** | **2021/22 £^3^** |
| Fit | 1628.35 | 1904.88 | 1866.07 |
| Mild | 2189.40 | 2561.21 | 2388.48 |
| Moderate | 2836.95 | 3318.73 | 2884.86 |
| Severe | 3736.55 | 4371.10 | 3823.14 |
| ^1^ Data source: Han et al (2019) [3]  ^2^ Data source for average NHS cost inflation between 2013 and 2019 of 1.98%: Curtis and Burns (2019) [4].  ^3^ Comorbidity healthcare costs are derived by subtracting the acute healthcare costs of falls from all-cause healthcare costs. Methods for estimating the acute healthcare costs are presented elsewhere (see Appendix B of [5]). | | | |

| **Table A4** Logistic regression for GP routine contact. | | |
| --- | --- | --- |
| ***Dependent variable: GP routine contact^1^ in ELSA Wave 5 (N=6,094)*** | | |
| **Explanatory variables** | **Coefficient (SE)^2^** | **P-value** |
| Constant | -20.065 (3.041) | <0.001 |
| Age W4 | 0.568 (0.085) | <0.001 |
| Age^2 W4 | -0.004 (0.0006) | <0.001 |
| Female | -0.192 (0.079) | 0.015 |
| Falls incidence W5 (ref: No fall incidence) |  |  |
| *Single non-MA fall* | 0.269 (0.127) | 0.034 |
| *Recurrent non-MA falls* | 0.491 (0.165) | 0.003 |
| *Single MA fall* | 0.973 (0.247) | <0.001 |
| *Recurrent falls with MA* | 0.933 (0.364) | 0.010 |
| Frailty W4 (0-100) | 0.132 (0.013) | <0.001 |
| Frailty^2 W4 | -0.002 (0.0003) | <0.001 |
| Change in frailty^3^ | 0.038 (0.008) | <0.001 |
| Cognitive impairment W4 | -0.692 (0.095) | <0.001 |
| Abnormal gait/balance W4 | -0.329 (0.121) | 0.007 |
| Social care receipt W4 | -0.785 (0.302) | 0.009 |
| Informal care receipt W4 | -0.255 (0.120) | 0.034 |
| GP routine contact^1^ W4 | 1.799 (0.081) | <0.001 |
| ^1^ A variable for whether the individual received a blood pressure check at the GP in the past 12 months was used.  ^2^ Coefficient greater than zero implies the explanatory variable increased the odds of the dependent variable relative to its reference level, and vice versa.  ^3^ Two-year change in frailty between ELSA W4 and W5.  **Abbreviation:** ELSA: English Longitudinal Study of Ageing; MA fall: fall requiring medical attention; Ref: reference; SE: standard error; W4: ELSA Wave 4; W5: ELSA Wave 5 | | |

| **Table A5** Logistic regression for self-referred exercise demand. | | |
| --- | --- | --- |
| ***Dependent variable: Self-referred exercise demand^1^ in ELSA Wave 5 (N=6,094)*** | | |
| **Explanatory variables** | **Coefficient (SE)^2^** | **P-value** |
| Constant | -3.211 (0.464) | <0.001 |
| Age W4 | -0.017 (0.006) | 0.010 |
| Female | 0.627 (0.096) | <0.001 |
| Falls incidence W5 (ref: No fall incidence) |  |  |
| *Single non-MA fall* | 0.184 (0.137) | 0.179 |
| *Recurrent non-MA falls* | 0.264 (0.148) | 0.075 |
| *Single MA fall* | 0.371 (0.191) | 0.052 |
| *Recurrent falls with MA* | 0.490 (0.212) | 0.021 |
| Frailty W4 (0-100) | 0.069 (0.015) | <0.001 |
| Frailty^2 W4 | -0.0008 (0.0003) | 0.013 |
| Change in frailty^3^ | 0.040 (0.007) | <0.001 |
| High physical activity W4 | 0.590 (0.115) | <0.001 |
| Abnormal gait/balance W4 | -0.253 (0.129) | 0.050 |
| OOP care receipt W4 | 0.480 (0.208) | 0.021 |
| Exercise receipt^1^ W4 | 1.812 (0.107) | <0.001 |
| ^1^ Variables for whether an individual is currently participating in an exercise or PT session were combined into a single variable and used.  ^2^ Coefficient greater than zero implies the explanatory variable increased the odds of the dependent variable relative to its reference level, and vice versa.  ^3^ Two-year change in frailty between ELSA W4 and W5.  **Abbreviation:** ELSA: English Longitudinal Study of Ageing; MA fall: fall requiring medical attention; OOP: out-of-pocket; Ref: reference; SE: standard error; W4: ELSA Wave 4; W5: ELSA Wave 5 | | |

# (2) Incorporating a wide range of societal outcomes

| **Table A6** Logistic regression for being in paid employment. | | |
| --- | --- | --- |
| ***Dependent variable: Paid employment^1^ in Wave 5 (N=6,205)*** | | |
| **Explanatory variables** | **Coefficient (SE)^2^** | **P-value** |
| Constant | 1.819 (0.703) | 0.010 |
| Age W4 | -0.066 (0.010) | <0.001 |
| Female | -0.220 (0.102) | 0.032 |
| Frailty W4 (0-100) | -0.043 (0.009) | <0.001 |
| Change in frailty^3^ | -0.035 (0.011) | 0.001 |
| Paid employment^1^ W4 | 3.757 (0.116) | <0.001 |
| ^1^ ELSA W4-5 contained information on whether the respondent was in paid employment in the previous week.  ^2^ Coefficient greater than zero implies the explanatory variable increased the odds of the dependent variable relative to its reference level, and vice versa.  ^3^ Two-year change in frailty between ELSA W4 and W5.  **Abbreviation:** ELSA: English Longitudinal Study of Ageing; MA fall: fall requiring medical attention; OOP: out-of-pocket; Ref: reference; SE: standard error; W4: ELSA Wave 4; W5: ELSA Wave 5 | | |

| **Table A7** Annual all-cause out-of-pocket care costs by SES quartile and frailty category. | | | | |
| --- | --- | --- | --- | --- |
| **Frailty/*SES quartile*** | ***Most privileged*** | ***2^nd^ quartile*** | ***3^rd^ quartile*** | ***Most deprived*** |
| Fit | £2,730.00^1^ | £2,730.00 | £2,730.00 | £0 |
| Mild | £3,726.45 | £3,336.06 | £2,912.91 | £2,730.00 |
| Moderate | £7,067.97 | £5,460.00 | £5,571.93 | £6,046.95 |
| Severe | £10,537.8 | £7,275.45 | £6,491.94 | £2,730.00 |
| ^1^ The distribution of OOP care visit per week varied by frailty category and SES quartile according to English Longitudinal Study of Ageing Wave 4 data. It was assumed 2.5 hours per visit, 52 weeks of visit per year, and cost of £21 per hour of visit.  **Abbreviation:** OOP: out-of-pocket; SES: socioeconomic status | | | | |

| **Table A8** Logistic regression for informal care receipt. | | |
| --- | --- | --- |
| ***Dependent variable: Informal care receipt^1^ in Wave 5 (N=6,205)*** | | |
| **Explanatory variables** | **Coefficient (SE)^2^** | **P-value** |
| Constant | -4.120 (0.161) | <0.001 |
| Female | 0.385 (0.076) | <0.001 |
| Frailty W4 (0-100) | 0.147 (0.013) | <0.001 |
| Frailty^2 W4 | -0.001 (0.0003) | <0.001 |
| Change in frailty^3^ | 0.104 (0.006) | <0.001 |
| Abnormal gait/balance W4 | 0.309 (0.095) | 0.001 |
| Social care receipt W4 | -0.657 (0.285) | 0.021 |
| OOP care receipt W4 | -0.526 (0.187) | 0.005 |
| Informal care receipt^1^ W4 | 1.389 (0.093) | <0.001 |
| Multiple informal care needs W4 | 0.724 (0.145) | <0.001 |
| ^1^ ELSA W4-5 contained information on the receipt of informal help for activities of daily living from non-professionals (e.g., partner, sibling, child).  ^2^ Coefficient greater than zero implies the explanatory variable increased the odds of the dependent variable relative to its reference level, and vice versa.  ^3^ Two-year change in frailty between ELSA W4 and W5.  **Abbreviation:** ELSA: English Longitudinal Study of Ageing; MA fall: fall requiring medical attention; Ref: reference; SE: standard error; W4: ELSA Wave 4; W5: ELSA Wave 5; OOP: out-of-pocket | | |

# References

1. Searle SD, Mitnitski A, Gahbauer EA, Gill TM, Rockwood K. A standard procedure for creating a frailty index. BMC geriatrics. 2008;8(1):24.

2. Clegg A, Bates C, Young J, Ryan R, Nichols L, Ann Teale E, et al. Development and validation of an electronic frailty index using routine primary care electronic health record data. Age Ageing. 2016;45(3):353-60. doi: 10.1093/ageing/afw039. PubMed PMID: 26944937; PubMed Central PMCID: PMCPMC4846793.

3. Han L, Clegg A, Doran T, Fraser L. The impact of frailty on healthcare resource use: a longitudinal analysis using the Clinical Practice Research Datalink in England. Age and Ageing. 2019;48(5):665-71.

4. Curtis LA, Burns A. Unit costs of health and social care 2019. PSSRU K, UK, editor2019.

5. Kwon J, Squires H, Young T. Economic model of community-based falls prevention: seeking methodological solutions in evaluating the efficiency and equity of UK guideline recommendations. BMC Geriatrics. 2023;(In print).
